# Supplementary material for: AlveoMPU: Bridging the Gap in Lung Model Interactions Using a Novel Alveolar Bilayer Film
Source: Polymers (Basel). 2024 May 23;16(11):1486. doi: 10.3390/polym16111486 (PMC11174738; doi:10.3390/polym16111486)
Supplement: Supplementary file 1 [file polymers-16-01486-s001.zip › polymers-2991025-supplementary.pdf]

Supplementary Materials

	AlveoMPU	Commercial membrane
**SEM image**	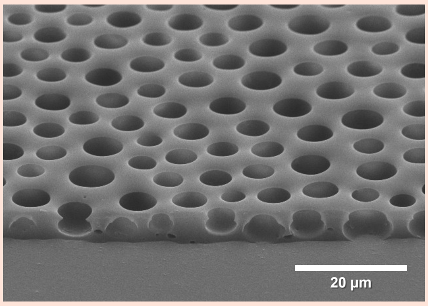	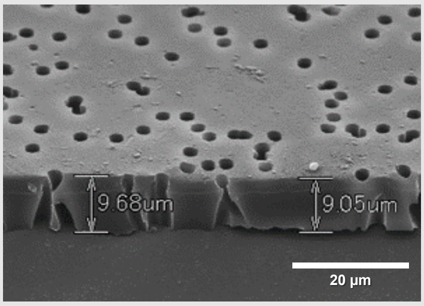
**Material**	**Foamed polyurethane**	**PC or PET**
**Thickness**	~ 5 µm	~ 10 µm
**Pore size**	A > B<br>~ 4–7 µm ~ 1–4 µm	A = B<br>(0.4, 1, 3, 5, 8 µm)
**Culture image**	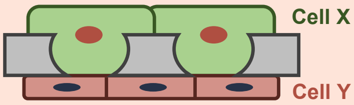	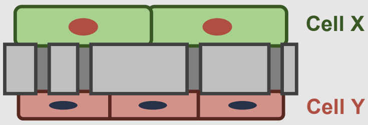

Figure S1. Comparison of scaffold characteristics of cell culture insert membranes between AlveoMPU and commercial porous membranes.

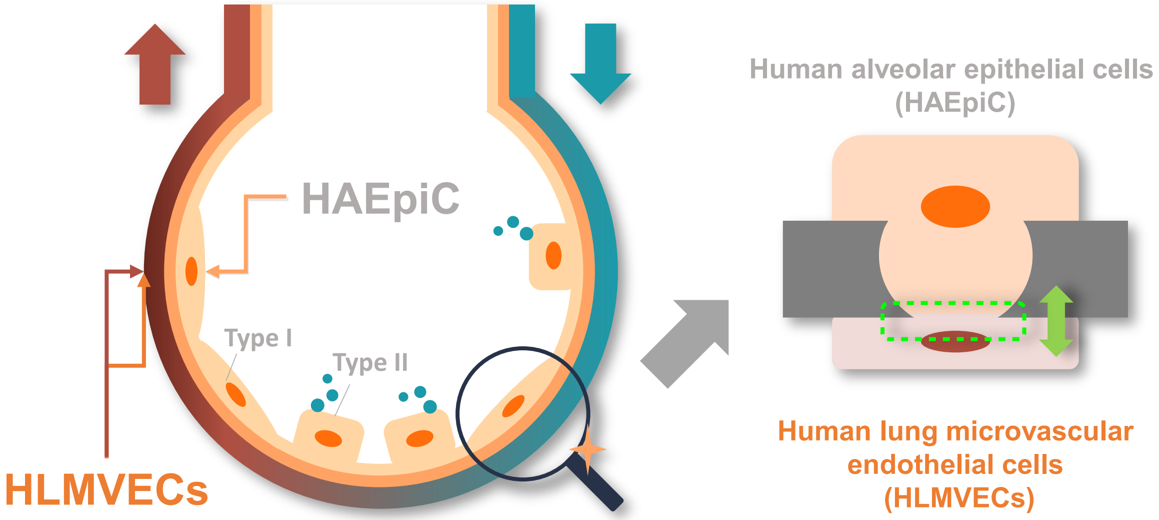

Figure S2. Reproduction of the 'field' of interaction between the two cell types that make up the alveoli using AlveoMPU.

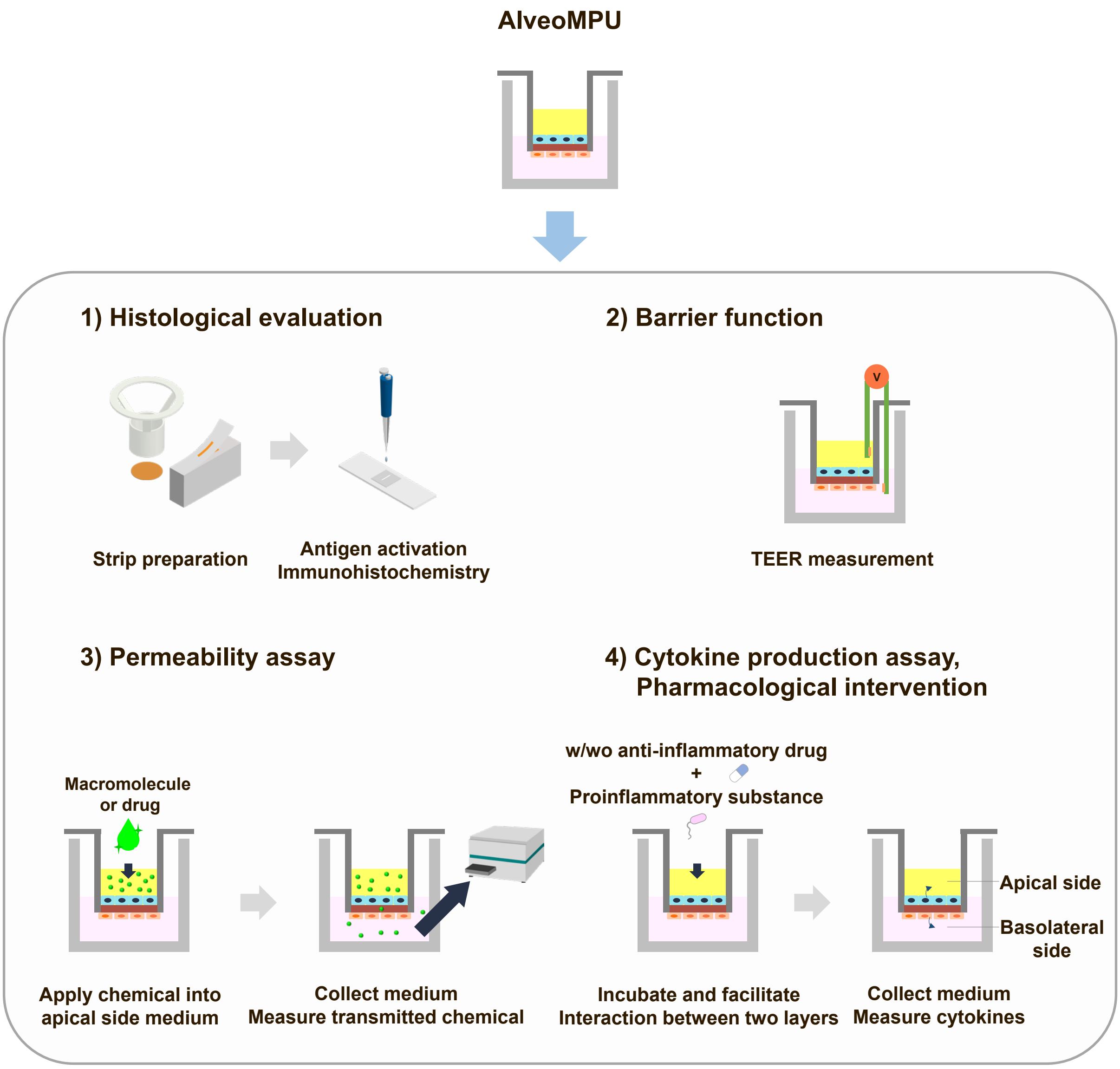

Figure S3. Examples of downstream applications of AlveoMPU.

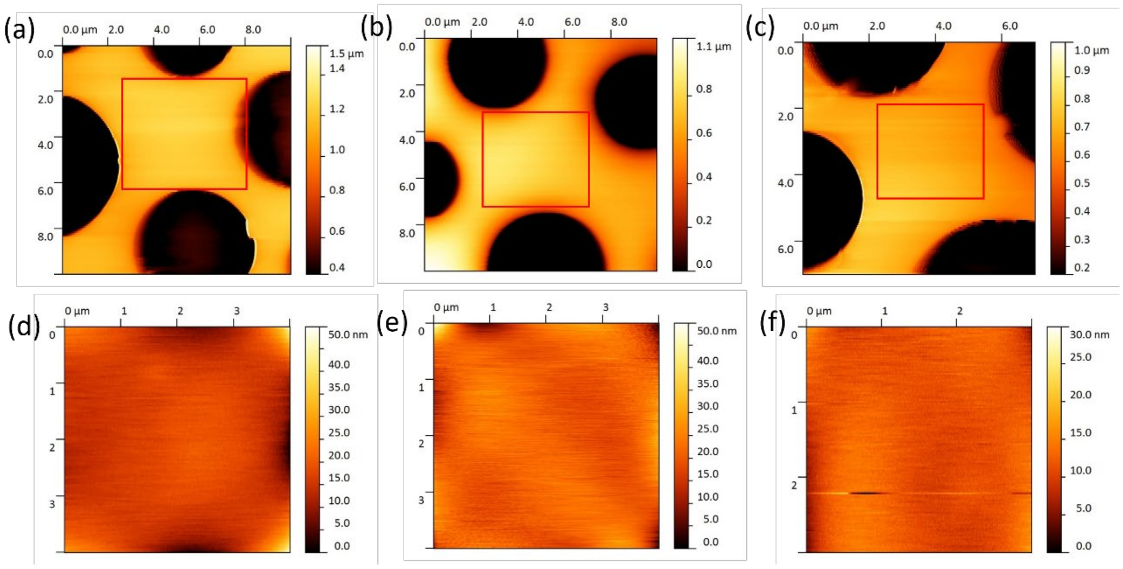

Figure S4. AFM height images. The images were corrected at three different points (a-c), and the synchronized data are shown in the corresponding column. The images in the lower row (d-f) were acquired by measuring the region marked with a red square in the images of the upper row (a-c).

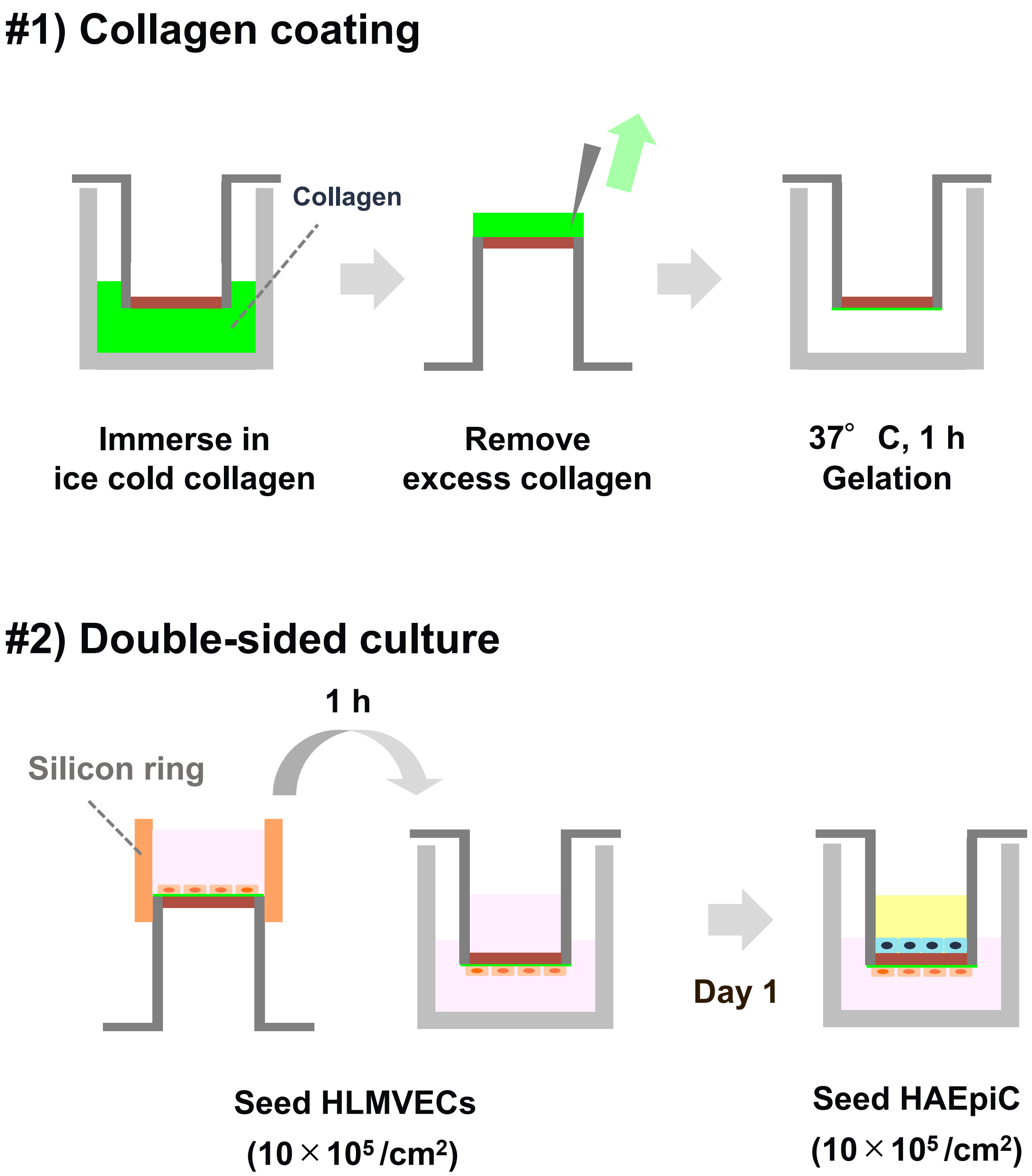

Figure S5. Schematic diagram showing AlveoMPU preparation using a porous polyurethane membrane.

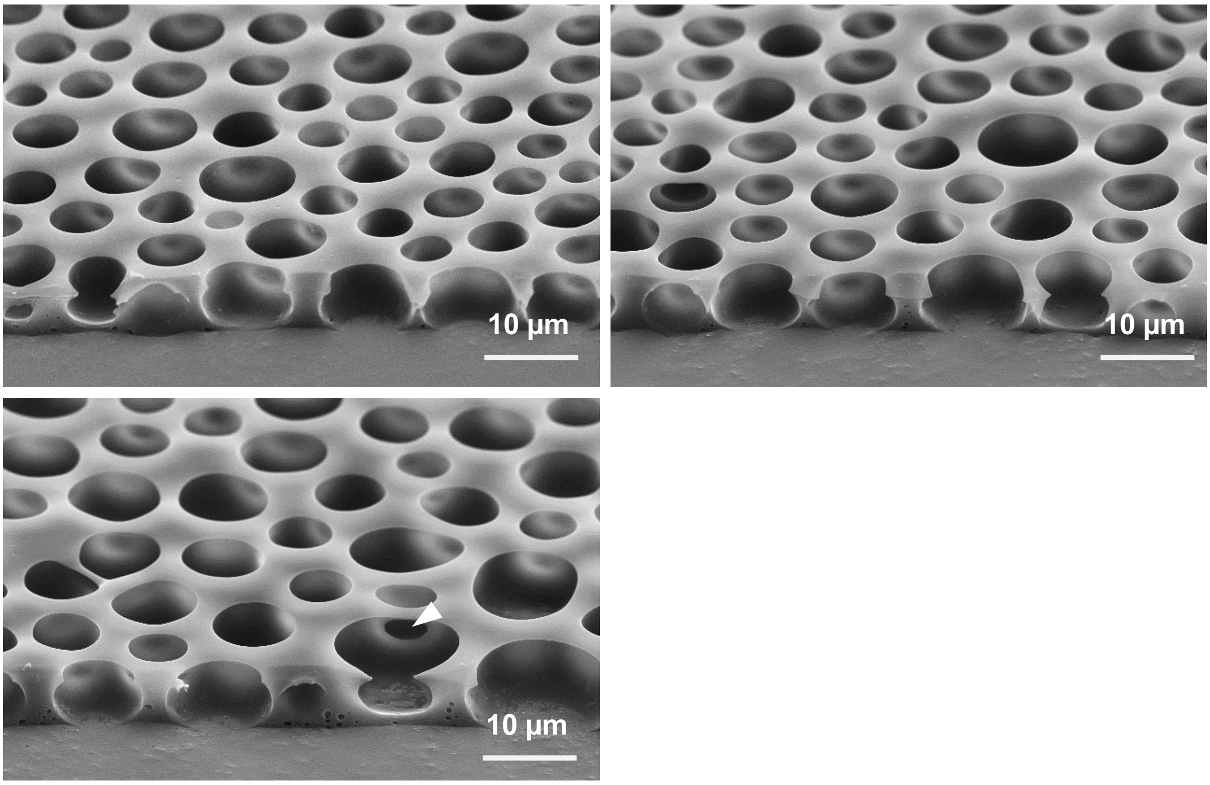

Figure S6. Representative scanning electron microscopy images viewed from an oblique angle. White arrowhead indicates the minor communicating path between adjacent pores.

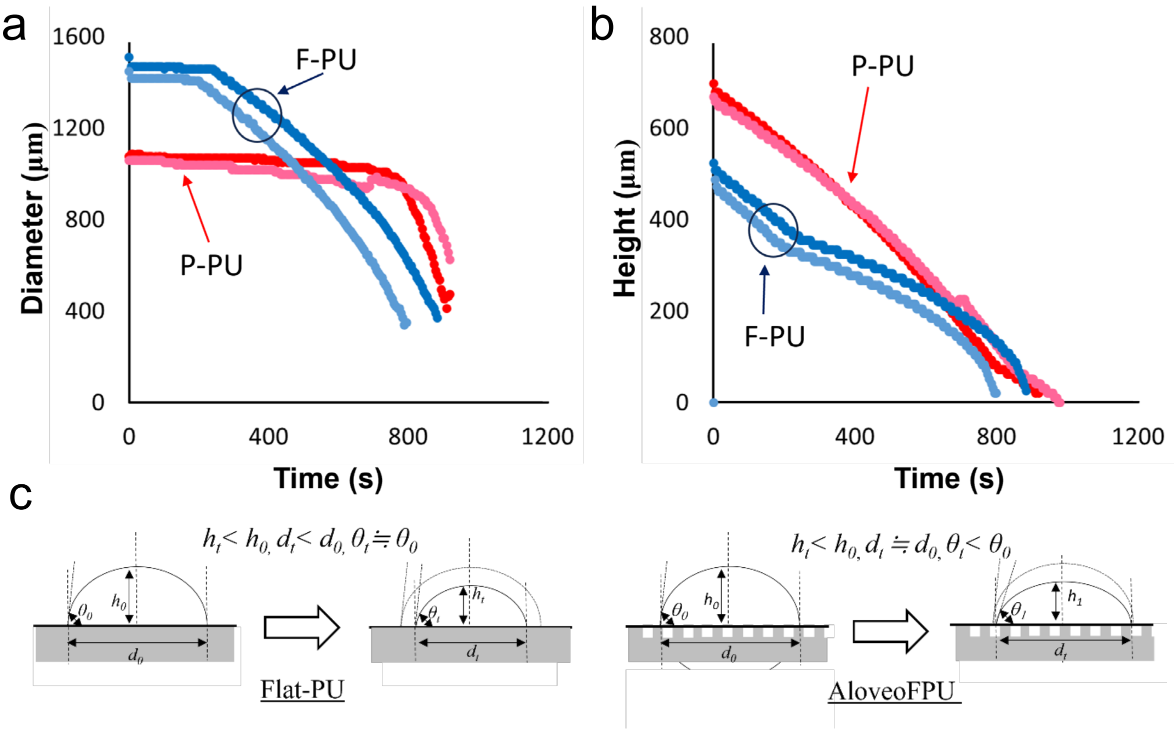

Figure S7. (a) Droplet diameter and (b) droplet height as functions of time. Both flat PU and porous PU used in AlveoMPU were analyzed for two independent samples each. (c) Schematic illustration showing the temporal change of a droplet on the membrane surfaces, where θ, d, and h represent contact angle, droplet diameter, and droplet height, respectively. Initial values (θ0, d0, h0) change with time (θt, dt, ht). Two independent samples for F-PU and P-PU were replicated and are denoted using blue and red curved lines, respectively.

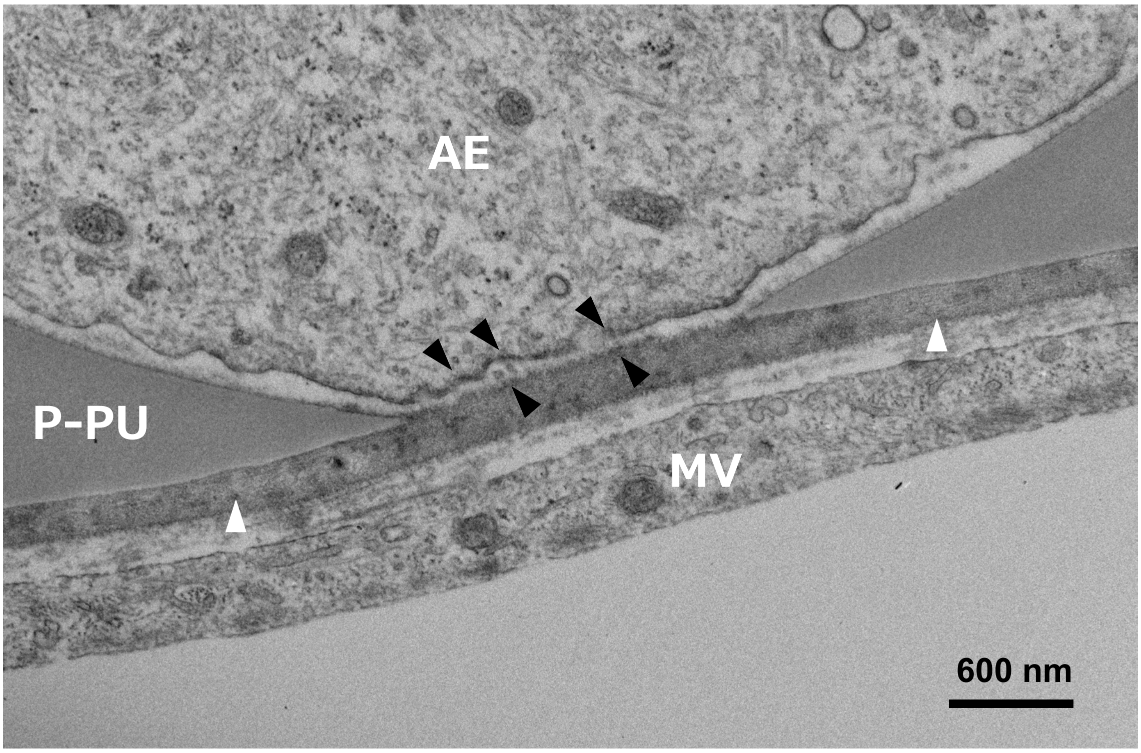

Figure S8. Ultrafine structure of the interaction region between alveolar and microvascular epithelial cells through the artificial basement membrane. AE: alveolar epithelial cell, MV: microvascular endothelial cell, c-(i) P-PU: porous polyurethane, white arrowhead: collagen thin layer (artificial basement membrane), black arrowhead: hemidesmosome-like structure.

Note S1: Additional discussions on surface roughness estimation using AFM

AFM observation was performed using a dynamic force mode in ambient air. A 20-μm scanner and a Si cantilever (SI-DF20) with a spring constant of 15.0 N/m was applied in all the measurements. Height images were collected with 512 × 256-pixel resolution and then characterized using the instrument-equipped software and Gwyddion software.

Figure SX(a-c) show AFM height images collected on the three different regions of the P-PU film. To evaluate the surface roughness of the P-PU film, height images were recorded on flat areas surrounding the pores (Figure SX(d-f)) of the film surface, marked in a red square in Figure SX (a-c). The obtained images were subjected to a first-order plane fit to remove sample tilt. The arithmetic (mean) average roughness (Ra) was employed to discuss the surface roughness. Ra was automatically calculated from the entire area of the height images shown in Figure SX(d-f). The obtained values on each image (Figure SX(d-f) were 2.28, 2.38, and 1.20 nm, respectively, and the averaged values were calculated to be 1.95 ± 0.53 nm. The low Ra value validates the smooth surface of the P-PU film.

Note S2: Additional discussions on surface wettability of the AlveoMPU membrane and the Flat-PU.

The time dependency of the droplet diameters and heights were plotted to gain insights into the surface wettability of the flat PU and AlveoMPU membranes (Figure S1). The droplet diameter on the flat PU film initially plateaued, followed by a swift and continuous decline (Figure S1a). Although the slope of the curve changed slightly when the droplet reached equilibrium, the droplet height continued to decrease over time (Figure S1b). For the AlveoMPU membrane, the droplet diameter remained unchanged, whereas its height steadily decreased over time. The observed phenomena may be attributed to the pinning effect, whereby the boundary of the droplet is anchored owing to surface roughness (Figure S1a, b) [1].

Based on these observations, we propose distinct dynamic contact angle models for the flat-PU and AlveoMPU films (Figure S1c). As shown in Figure 2e, the water contact angle of the flat PU film initially decreased and then remained almost constant. Upon reaching equilibrium, the water droplet gradually contracted owing to reductions in both its diameter and height, which made the contact angles stable (Figure S1c, left). For the AlveoMPU films, the droplet diameters were pinned until they significantly decreased (Figure S1a). Because of the pinning effect and water infiltration into the pores via capillary condensation, the droplet contact angles exhibited a relatively rapid decline with time. In this model, a decrease in droplet height was responsible for the reduction in the contact angles (Figure S1c, right).

<Reference>

(1) Suzuki, S,; Ueno, K. Apparent contact angle calculated from a water repellent model with pinning effect. *Langmuir*. **2017,** *44*, 138–143. DOI:10.1021/acs.langmuir.6b03832

Supplementary Table 1

Type	Antibody Name	Supplier	Cat. No.	Target
Primary antibody	Rabbit monoclonal anti-vimentin	Nichirei Bioscience	413541	Vimentin
Secondary antibody	Goat anti-Rabbit IgG Alexa Fluor™ 555	Invitrogen	A21202	
Primary antibody	Anti-pan Cytokeratin antibody [AE1+AE3]	abcam	ab961	Cytokeratin AE1/AE3
Secondary antibody	Donkey anti-Mouse IgG Alexa Fluor™ 488	Invitrogen	A21428	
Primary antibody	Mouse Occludin monoclonal antibody (OC-3F10)	Invitrogen	40-2200	Occludin
Secondary antibody	Goat anti-Mouse IgG Alexa Fluor™ 488	Invitrogen	A11001	
Primary antibody	ZO-1 polyclonal antibody	Invitrogen	33-1500	ZO-1
Secondary antibody	Goat anti-Rabbit IgG Alexa Fluor™ 546	Invitrogen	A11010	
